# Enhanced adherence of methicillin-resistant *Staphylococcus pseudintermedius* sequence type 71 to canine and human corneocytes

**DOI:** 10.1186/1297-9716-45-70

**Published:** 2014-06-24

**Authors:** Francesca Latronico, Arshnee Moodley, Søren Saxmose Nielsen, Luca Guardabassi

**Affiliations:** 1 Department of Veterinary Disease Biology, Faculty of Health and Medical Sciences, University of Copenhagen, 1870 Frederiksberg C, Denmark; 2 Department of Large Animal Sciences, Faculty of Health and Medical Sciences, University of Copenhagen, 1870 Frederiksberg C, Denmark

## Abstract

The recent worldwide spread of methicillin-resistant *Staphylococcus pseudintermedius* (MRSP) in dogs is a reason for concern due to the typical multidrug resistance patterns displayed by some MRSP lineages such as sequence type (ST) 71. The objective of this study was to compare the *in vitro* adherence properties between MRSP and methicillin-susceptible (MSSP) strains. Four MRSP, including a human and a canine strain belonging to ST71 and two canine non-ST71 strains, and three genetically unrelated MSSP were tested on corneocytes collected from five dogs and six humans. All strains were fully characterized with respect to genetic background and cell wall-anchored protein (CWAP) gene content. Seventy-seven strain-corneocyte combinations were tested using both exponential- and stationary-phase cultures. Negative binomial regression analysis of counts of bacterial cells adhering to corneocytes revealed that adherence was significantly influenced by host and strain genotype regardless of bacterial growth phase. The two MRSP ST71 strains showed greater adherence than MRSP non-ST71 (*p* < 0.0001) and MSSP (*p* < 0.0001). This phenotypic trait was not associated to any specific CWAP gene. In general, *S. pseudintermedius* adherence to canine corneocytes was significantly higher compared to human corneocytes (*p* < 0.0001), but the MRSP ST71 strain of human origin adhered equally well to canine and human corneocytes, suggesting that MRSP ST71 may be able to adapt to human skin. The genetic basis of the enhanced *in vitro* adherence of ST71 needs to be elucidated as this phenotypic trait may be associated to the epidemiological success and zoonotic potential of this epidemic MRSP clone.

## Introduction

*Staphylococcus pseudintermedius* is the most common bacterial pathogen associated with otitis and pyoderma in dogs, which are the natural hosts of this staphylococcal species. The reported carriage prevalences in healthy dogs range between 46 and 92% depending on the study population and methodology used for assessing carriage [1]. Methicillin-resistant *S. pseudintermedius* (MRSP) were first reported in the United States in 1999 [2] and in 2007 in Europe [3]. An increasing number of studies have documented the rapid spread of MRSP worldwide [4-8]. Some strains are resistant to all antibiotics available for treatment in small animal practice and two dominant clonal lineages have been recognized: ST71 in Europe and ST68 in North America [6]. It has been demonstrated that MRSP ST71 isolates are better biofilm producers compared to other MRSP clones [9]. Although the ability to form biofilm may play an important role in the pathophysiology of bacterial infections and be related to survival and persistence of *S. pseudintermedius* in the environment [9,10], the reasons for the rapid emergence and success of this lineage remain unknown.

Recent studies have documented carriage of MRSP among veterinary staff and pet owners [11-14]. Sporadic cases of human infection have also been reported, including patients exposed to colonized household pets [15-17]. Staphylococcal adherence to corneocytes is the first important step to skin colonization. *S. aureus* adherence to skin and mucosae results from the interaction between host extracellular matrix proteins, such as fibrinogen, fibronectin and cytokeratin 10, and bacterial cell wall-associated proteins (CWAPs) termed microbial surface components recognizing adhesive matrix molecules (MSCRAMMs) [18]. Eighteen CWAP genes have been described in *S. pseudintermedius*[19], and are hypothesized to play a role in pathogenesis [20,21].

To shed light on the epidemiological success and zoonotic potential of MRSP, the *in vitro* adherence properties of four MRSP strains belonging to three unrelated sequence types (ST71, ST68 and ST258) were compared to those of three genetically unrelated methicillin-susceptible (MSSP) strains (ST25, ST257, ST259) using corneocytes obtained from five dogs and six humans. The CWAP gene profiles of all strains were determined to identify possible associations with adherence ability.

## Materials and methods

### Strains

The four MRSP and three MSSP strains used in this study were isolated from different body sites of healthy and diseased dogs and from the nostrils of healthy humans (Table 1). All strains were typed by the new seven genes multilocus sequence typing scheme (MLST) [22], *spa* typing [6], and pulsed-field gel electrophoresis (PFGE) [23]. The MRSP strains were selected to represent the two epidemic clones ST71 and ST68 [6], and ST258 (ST106 according to the old MLST scheme), which is an emerging clone in the Scandinavian countries [9,24]. MRSP ST71 was represented by two strains of canine and human origin. ED99, a whole genome sequenced strain [25] and two randomly selected strains from a healthy dog [23] and a dog owner [26] were used as MSSP strains. For all strains, the presence of genes encoding 18 putative CWAPs from *SpsA* to *SpsR* was determined by PCR using strain ED99 as a positive control as described by Bannoehr et al. [19].

**Table 1:** Strains used in this study

**Strain**	**MSSP/MRSP**	**ST**	**Host origin**	**Specimen**	***spa***	***CWAPs***				
						***SpsD***	***SpsF***	***SpsO***	***SpsP***	***SpsQ***
**E140**	MRSP	71	Dog	Wound clinical	t02	+	+	-	+	+
**Franca 29a**	MRSP	71	Human	Nose commensal	t02	+	+	-	+	+
**USA 06-3228**	MRSP	68	Dog	Skin clinical	t06	+	+	-	+	+
**E139**	MRSP	258	Dog	Ear clinical	t02	-	-	-	-	-
**ED99**	MSSP	25	Dog	Skin clinical	t01	+	+	+	+	+
**2M**	MSSP	257	Dog	Mouth commensal	n.t.	+	+	+	-	-
**Eva 70**	MSSP	259	Human	Nose commensal	n.t.	-	-	-	-	-

MSSP: methicillin-susceptible *S. pseudintermedius*; MRSP: methicillin-resistant *S. pseudintermedius*; ST: sequence type; *spa*: *spa* type; CWAPs: genes encoding putative cell-wall anchored proteins; n.t. = non-typeable.

### Collection of corneocytes

Corneocytes were collected from five dogs of different breeds (one Rottweiler, one Parson Jack Russel Terrier, one Springel Spaniel and two Shetland Sheepdog; age range: 6–10 years) and from six humans (age range: 30–35 years). The dogs were healthy with no symptoms of skin or ear infection, and had no history of systemic or local antimicrobial therapy in the last six months before sampling. None of the human donors had a history of skin disorder or reported to have frequent contacts with dogs. Written informed consent was obtained for each canine and human donor prior to sampling. Corneocytes were collected by applying adhesive discs of 25-mm diameter (D-squame, CuDerm Corporation, Dallas, USA) on the canine skin of the inner concave side of the pinna and on the human inner forearm [27,28]. Twenty-eight discs with confluent corneocytes layer were obtained from each donor at the same time to avoid sampling variability. Before collection, surface debris was removed by applying five successive adhesive tape strips (Sellotape® Original, Winsford, Cheshire, UK). After collection, the adhesive discs were examined by light microscopy to evaluate the confluency of the corneocytes layer, and then stored at 4 °C for a maximum of 10 months. At the time of collection, all donors were screened for *S. pseudintermedius*. Canine oral and perineal swabs and human nasal swabs were collected and processed as described before [12,23].

### *In vitro* corneocytes adherence assay

All experiments were carried out using bacterial cultures in mid-exponential and late stationary phase. Briefly, each strain was inoculated into 10 mL BHI and incubated at 37 °C for 14–16 h (late stationary phase culture). After the overnight incubation, 100 μL of bacterial culture was transferred into 30 mL BHI and incubated at 37 °C until optical density (OD)₆₀₀ = 0.750 ± 0.030 (mid-exponential phase culture). Thereafter, 8 mL were centrifuged at 1500 *g* for 5 min at 4 °C, and the resulting pellet was washed three times with phosphate buffered saline (PBS, Dulbecco’s Phosphate-buffered saline no calcium no magnesium, GIBCO) by pipetting and centrifugation (800 *g* for 3 min). The final bacterial suspension was adjusted to an OD₆₀₀ = 0.15, which corresponds to ~ 7 × 10^7^ CFU/mL in stationary phase and ~ 3 × 10^7^ CFU/mL in exponential phase. Subsequently, 500 μL of OD-adjusted suspension was pipetted onto D-squame discs with adhered corneocytes to form a meniscus and incubated at 37 °C for 45 min in a moist chamber. After incubation, the discs were washed with PBS for three times (3 × 10 s) and air dried. Discs were stained for one minute with 500 μL of 0.5% crystal violet and washed with PBS to remove excess stain. The number of adherent bacteria was counted at × 1000 magnification using an Axioplan II epifluorescence microscope (Zeiss, Oberkochen, Germany) and a Zeiss AxioCam digital camera. Ten microscopic field images with confluent corneocytes were randomly selected, except for dog D2 with strain 2M in stationary phase where it was possible to use only three fields due to poor confluent corneocyte layer. One slide for each combination of strain, growth phase (mid-exponential or stationary) and corneocytes donor (dog or human) was used. To assess the reproducibility of the results in our setting, a pilot experiment was performed where the combinations of each strain in stationary phase and human corneocytes were tested in duplicate. One corneocytes disc per individual was incubated with PBS only and used as a negative control.

### Statistical analyses

The mean counts for all combinations of three strain genotypes (MRSP ST71, MRSP non-ST71 and MSSP), two growth phases (mid-exponential and stationary), two strain host origins (dog or human) and two corneocytes donor types (dog or human), were compared in a negative binomial regression model using the Genmod procedure in SAS v. 9.3 (SAS Institute, Cary, NC, USA). All ten replicates within each donor were treated as repeated samples within a donor in the model. This model was used to compare adherence of *S. pseudintermedius* to corneocytes among the different strain genotypes, the strain host origin, the corneocytes donor origin and the *S. pseudintermedius* colonization status of the donor at the time of the collection of corneocytes while stratifying in the exponential and the stationary phase. A *p*-value of < 0.05 was deemed significant.

## Results

**Figure 1:**
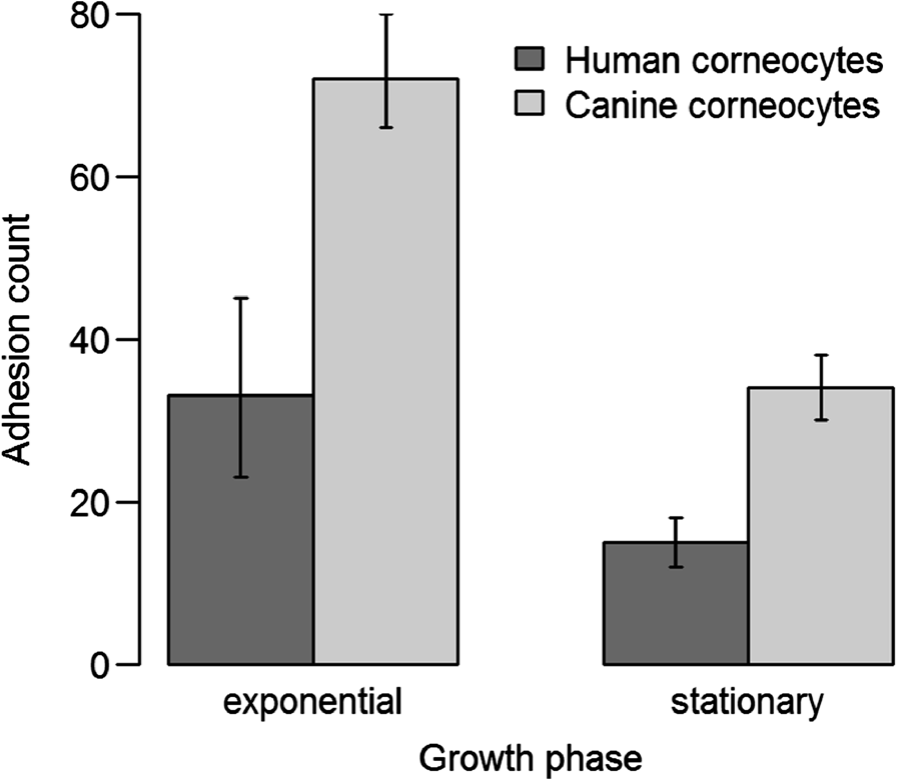
**Overall mean adhesion counts of all *Staphylococcus pseudintermedius *strains to human and canine corneocytes.** Error bars indicate 95% confidence intervals.

**Figure 2:**
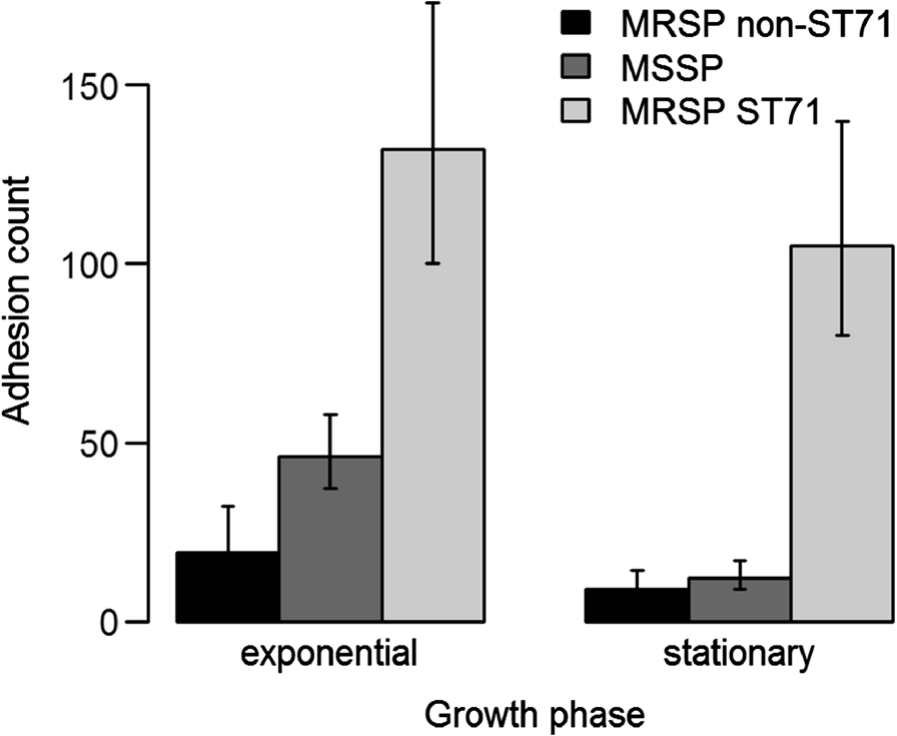
**Overall mean adhesion counts of MRSP ST71, MRSP non-ST71 and MSSP to corneocytes of human and canine origin.** Error bars indicate 95% confidence intervals.

**Figure 3:**
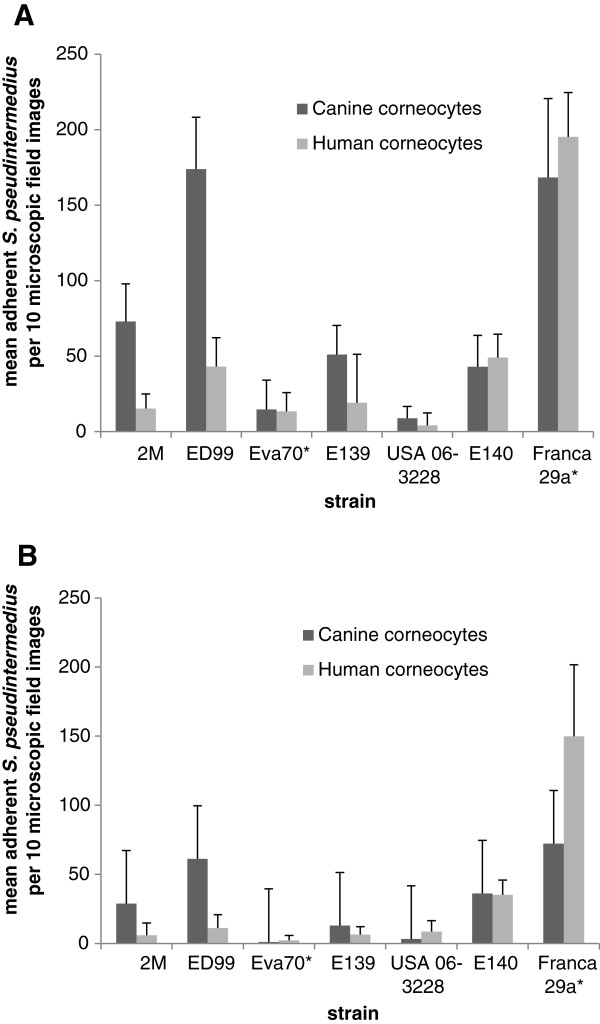
**Mean adhesion counts of the seven *Staphylococcus pseudintermedius *strains to human and canine corneocytes. (A)** Exponential phase. **(B)** Stationary phase. Bars represent the mean counts of adherent *Staphylococcus pseudintermedius* per 10 microscopic field images and the error bars indicate the 95% confidence intervals. The two strains of human origin are marked by an asterisk.

Four out of five canine corneocyte donors were positive for *S. pseudintermedius*, whereas all six human donors were negative. No statistically significant difference was found between colonization status at the time of the collection of corneocytes and adherence. Mean bacterial counts were affected by host (Figure 1) and strain genotype (Figure 2). Overall, *S. pseudintermedius* showed significantly greater adherence to canine corneocytes than to human corneocytes (*p* < 0.0001) (Figure 1). Preferential adherence to canine corneocytes was particularly evident for two MSSP isolated from dogs, ED99 and 2M (Figure 3). None of the strains showed a statistically significant preferential adherence to human corneocytes but the MRSP ST71 strain of human origin was the best adhering strain to both canine and human corneocytes (Figure 3), with double number of adhering bacteria to human corneocytes (mean count of all six human corneocytes = 150) compared to canine corneocytes (mean count of all five canine corneocytes = 72) in stationary phase (Figure 3B). In general, the mean adherent count of each strain was higher in mid-exponential phase than in stationary phase (Figure 3) and significant differences in adherence ability were observed between mean counts of MRSP and MSSP in stationary phase (*p* < 0.001) but not in the exponential phase (*p* = 0.05). Interestingly, MRSP ST71 showed greater adherence than MRSP non-ST71 and MSSP (*p* < 0.0001) regardless of growth phase (Figure 2). Strain host origin and the possible interactions among all investigated parameters were not significant. No bacteria were observed in the negative control corneocytes discs, confirming that corneocytes were not contaminated with strains from the donor after removal of surface debris prior to the corneocytes collection. No statistically significant differences were found between the mean bacterial counts of the two slides used to assess the reproducibility of the results in the pilot experiment.

All seven strains were positive for 13/18 CWAP genes (namely *spsA*-*C*, *spsE*, *spsG*, *spsH*, *spsI*-*N*, *spsR*). The remaining five genes; *spsD*, *spsF*, *spsO* and the *S. aureus spa* orthologues *spsP* and *spsQ* were present in different combinations (Table 1). Strain adherence was not associated to any specific CWAP gene combination. The *SpsO* sequence detected in 2M was 105 bp smaller compared to the expected size and showed 97% nucleotide identity with *SpsO* ED99.

## Discussion

We evaluated the adherence properties of MRSP and MSSP, showing that MRSP ST71 adhered better to canine and human corneocytes than MRSP non-ST71 and MSSP. The enhanced adherence of ST71 compared to other genotypes may explain the epidemiological success of this clone, which was first detected in Europe in 2007 [3] and then rapidly spread within this continent as well as in countries outside Europe such as Brazil, USA and Canada [6,29]. MRSP ST71 has recently been shown to have a greater ability to produce biofilm compared with other STs [9,10]. Taken together, these data indicate that this clone has a particular ability to adhere to skin and form biofilm compared to other members of the species, even though the genetic factors associated with these phenotypic traits remain to be identified.

All seven strains were tested using both mid-exponential and stationary phase because it has previously been shown in *S. aureus*, that cell wall adhesins and secreted virulence factors are differentially expressed during the bacterial growth cycle [30,31]. Adhesins are hypothesized to be predominantly expressed in exponential phase and at low cell densities, while toxins and other secreted virulence factors are expressed in post exponential phase and at high cell densities for host tissue invasion and dissemination [30,31]. As expected, all *S. pseudintermedius* had greater mean adherence counts in the mid-exponential phase compared to the stationary phase following the time-dependent expression pattern regulated by *agr* system in other staphylococcal species [32]. Interestingly, when analyzing MRSP vs MSSP, statistical differences were observed only in stationary phase. This observation could be a result of the differences between groups in the protein expression patterns and persistence of surface adhesins. Such differences were recently reported for the *S. aureus* surface adhesion proteins SdrD and SdrE [32] which have a biphasic expression pattern, and for clumping factor B and fibronectin-binding proteins (FnBPs) [33], which can be present throughout the bacterial growth cycle.. Georghean et al. [33] showed that high-level expression of FnBPs throughout the bacterial growth cycle is requested to promote the accumulation phase of *ica*-indipendent biofilm formation in methicillin-resistant *S. aureus*[33] and this may be also the case in MRSP.

*S. pseudintermedius* have significantly greater adherence to canine corneocytes than to human corneocytes, as indicated by previous studies [27,34]. Surprisingly, we found that the MRSP ST71 isolated from a small animal dermatologist in Italy [12] was the best strain adhering to both canine and human corneocytes. This finding suggests that MRSP ST71 may be able to adapt to human skin, as supported by the frequent reports of this lineage among veterinarians and pet owners [12,13], and in human infections [17]. The possible extended host spectrum of this lineage is indirectly suggested by the increasing number of reports of MRSP ST71 isolated from other animal species other than *Canidae* such as horses [7], donkeys [7], cats [7,35], cattle [36], and parrots [7]. Similarly, a broad host range has been hypothesized for some methicillin-resistant *S. aureus* clones of animal origin such as ST398 [37].

We screened all seven strains used in the *in vitro* assay by PCR for the 18 putative CWAPs previously described by Bannoehr et al. [19] to identify any specific CWAP or combinations of CWAPs that may explain any differences in the adherence ability observed for the different strains. We did not observe a clear association between the presence of any particular CWAP or a combination of them and the enhanced adherence to corneocytes. For example, the MRSP ST68 strain, USA 06–3228, and the MRSP ST71 strains, E140 and Franca 29a, had the same CWA surface proteins profile, but exhibited great variability in the adherence ability to canine and human corneocytes. However it should be noted that we only PCR screened for the presence of these CWAP genes and adherence may be related to differential CWA protein expression that could be affected at the post-transcriptional and post-translational level or by degradation proteases [32]. In addition, the 18 putative CWAPs were identified based on *in silico* analysis of only one methicillin-susceptible *S. pseudintermedius* strain genome (ED99). Our strains may contain other CWAPs not present in ED99 or the adherence to canine corneocytes may be mediated by non-proteinaceous adhesins like wall teichoic acids, as shown for *S. aureus* adherence to nasal epithelial cells [38].

*S. pseudintermedius* adherence did not differ significantly between the corneocytes collected from dog testing negative for *S. pseudintermedius* and those collected from the four positive dogs. This result is apparently in contrast with the findings of a previous study, where it was demonstrated that *in vitro* adherence of *S. pseudintermedius* to canine corneocytes is influenced by the carrier status of corneocyte donors [39]. However, this is only an apparent contrast since we could only determine whether the donors were positive at the time of sampling. Determination of their actual carrier status would have required a longitudinal study setup [23,39]. For example, the dog that tested negative for *S. pseudintermedius* at the time of sampling was not necessarily a non-carrier and could be sporadic or intermittent carrier according to the classification proposed by Paul et al. [23].

There are two major limitations in this study. First, the limited number of strains used in this study may reflect strain-dependent than group-dependent factors. Unfortunately it is not feasible to perform the adherence corneocytes assay using a larger number of strains due to the limited number of corneocytes adhesive discs that can be collected from a single donor at the same time. Second, our results are based on the mean adherence of ten different microscopic field images, and biological replicates from the same donor were not performed. However, “donor” was not a unit of concern and the corneocytes adherence assay was shown to be reproducible by the pilot experiment as well as by previous studies [27,28].

In conclusion, under *in vitro* conditions, MRSP ST71 adhered better to corneocytes compared to MRSP non-ST71 and MSSP. The enhanced adherence of ST71 may be a factor contributing to the epidemiological success of this MRSP lineage. Another notable finding was the remarkable ability of a human isolate of MRSP ST71 to adhere to human corneocytes, which confirms the hypothesis that this lineage may have higher ability to adapt to humans and ultimately possess extended host range and higher zoonotic potential compared to other lineages of the bacterial species [12]. Further studies using a combined approach based on whole genome sequencing, proteomics and functional assays are needed to elucidate the genetic factors responsible for the enhanced *in vitro* adherence of MRSP ST71.

## Competing interests

The authors declare that they have no competing interests.

## Authors’ contributions

Conceived and designed the experiments: FL, AM, LG. Performed the experiments: FL. Analyzed the data: FL, SSN. Contributed reagents/materials/analysis tools: FL, SSN, LG. Wrote and approved the final manuscript: FL, AM, SSN, LG.

## References

[B1] Bannoehr J Guardabassi L *Staphylococcus pseudintermedius* in the dog: taxonomy, diagnostics, ecology, epidemiology and pathogenicity Vet Dermatol 2012 23 253 266 22515504 10.1111/j.1365-3164.2012.01046.x

[B2] Gortel K Campbell KL Kakoma I Whittem T Schaeffer DJ Weisiger RM Methicillin resistance among staphylococci isolated from dogs Am J Vet Res 1999 60 1526 1530 10622162

[B3] Loeffler A Linek M Moodley A Guardabassi L Sung JM Winkler M Weiss R Lloyd DH First report of multiresistant, mecA-positive *Staphylococcus intermedius* in Europe: 12 cases from a veterinary dermatology referral clinic in Germany Vet Dermatol 2007 18 412 421 17991158 10.1111/j.1365-3164.2007.00635.x

[B4] Bardiau M Yamazaki K Ote I Misawa N Mainil JG Characterization of methicillin-resistant *Staphylococcus pseudintermedius* isolated from dogs and cats Microbiol Immunol 2013 57 496 501 23607810 10.1111/1348-0421.12059

[B5] Feng Y Tian W Lin D Luo Q Zhou Y Yang T Deng Y Liu YH Liu JH Prevalence and characterization of methicillin-resistant *Staphylococcus pseudintermedius* in pets from South China Vet Microbiol 2012 160 517 524 22770517 10.1016/j.vetmic.2012.06.015

[B6] Perreten V Kadlec K Schwarz S Grönlund Andersson U Finn M Greko C Moodley A Kania SA Frank LA Bemis DA Franco A Iurescia M Battisti A Duim B Wagenaar JA van Duijkeren E Weese JS Fitzgerald JR Rossano A Guardabassi L Clonal spread of methicillin-resistant *Staphylococcus pseudintermedius* in Europe and North America: an international multicentre study J Antimicrob Chemother 2010 65 1145 1154 20348087 10.1093/jac/dkq078

[B7] Ruscher C Lübke-Becker A Semmler T Wleklinski CG Paasch A Soba A Stamm I Kopp P Wieler LH Walther B Widespread rapid emergence of a distinct methicillin- and multidrug-resistant *Staphylococcus pseudintermedius* (MRSP) genetic lineage in Europe Vet Microbiol 2010 144 340 346 20181441 10.1016/j.vetmic.2010.01.008

[B8] Wang Y Yang J Logue CM Liu K Cao X Zhang W Shen J Wu C Methicillin-resistant *Staphylococcus pseudintermedius* isolated from canine pyoderma in North China J Appl Microbiol 2012 112 623 630 22229826 10.1111/j.1365-2672.2012.05233.x

[B9] Osland AM Vestby LK Fanuelsen H Slettemeås JS Sunde M Clonal diversity and biofilm-forming ability of methicillin-resistant *Staphylococcus pseudintermedius* J Antimicrob Chemother 2012 67 841 848 22258925 10.1093/jac/dkr576

[B10] Singh A Walker M Rosseau J Weese JS Characterization of the biofilm forming ability of *Staphylococcus pseudintermedius* from dog BMC Vet Res 2013 9 93 23641755 10.1186/1746-6148-9-93 PMC3681638

[B11] Morris DO Boston RC O’Shea K Rankin SC The prevalence of carriage of meticillin-resistant staphylococci by veterinary dermatology practice staff and their respective pets Vet Dermatol 2010 21 400 407 20409076 10.1111/j.1365-3164.2010.00866.x

[B12] Paul NC Moodley A Ghibaudo G Guardabassi L Carriage of methicillin-resistant *Staphylococcus pseudintermedius* in small animal veterinarians: indirect evidence of zoonotic transmission Zoonoses Public Health 2011 58 533 539 21824350 10.1111/j.1863-2378.2011.01398.x

[B13] Laarhoven LM de Heus P van Luijn J Duim B Wagenaar JA van Duijkeren E Longitudinal study on methicillin-resistant *Staphylococcus pseudintermedius* in households PLoS One 2011 6 e27788 22132141 10.1371/journal.pone.0027788 PMC3223215

[B14] Van Duijkeren E Kamphuis M van der Mije IC Laarhoven LM Duim B Wagenaar JA Houwers DJ Transmission of methicillin-resistant *Staphylococcus pseudintermedius* between infected dogs and cats and contact pets, humans and the environment in households and veterinary clinics Vet Microbiol 2011 150 338 343 21420256 10.1016/j.vetmic.2011.02.012

[B15] Kempker R Mangalat D Kongphet-Tran T Eaton M Beware of the pet dog: a case of *Staphylococcus intermedius* infection Am J Med Sci 2009 338 425 427 19826243 10.1097/MAJ.0b013e3181b0baa9

[B16] Savini V Barbarini D Polakowska K Gherardi G Bialecka A Kasprowicz A Polilli E Marrollo R Di Bonaventura G Fazii P D'Antonio D Miedzobrodzki J Carretto E Methicillin-resistant *Staphylococcus pseudintermedius* infection in a bone marrow transplant recipient J Clin Microbiol 2013 51 1636 1638 23486715 10.1128/JCM.03310-12 PMC3647946

[B17] Stegmann R Burnens A Maranta CA Perreten V Human infection associated with methicillin-resistant *Staphylococcus pseudintermedius* ST71 J Antimicrob Chemother 2010 65 2047 2048 20601356 10.1093/jac/dkq241

[B18] Clarke SR Foster SJ Surface adhesins of *Staphylococcus aureus* Adv Microb Physiol 2006 51 187 224 17010697 10.1016/S0065-2911(06)51004-5

[B19] Bannoehr J Ben Zakour NL Reglinski M Inglis NF Prabhakaran S Fossum E Smith DG Wilson GJ Cartwright RA Haas J Hook M van den Broek AH Thoday KL Fitzgerald JR Genomic and surface proteinomic analysis of the canine pathogen *Staphylococcus pseudintermedius* reveals proteins that mediate adherence to the extracellular matrix Infect Immun 2011 79 3074 3086 21576333 10.1128/IAI.00137-11 PMC3147560

[B20] Bannoehr J Brown JK Shaw DJ Fitzgerald RJ van den Broek AH Thoday KL *Staphylococcus pseudintermedius* surface proteins SpsD and SpsO mediate adherence to ex vivo canine corneocytes Vet Dermatol 2012 23 119 124 22112246 10.1111/j.1365-3164.2011.01021.x

[B21] Pietrocola G Geoghegan JA Rindi S Di Poto A Missineo A Consalvi V Foster TJ Speziale P Molecular characterization of the multiple interactions of SpsD, a surface protein from *Staphylococcus pseudintermedius*, with host extracellular matrix proteins PLoS One 2013 8 e66901 23805283 10.1371/journal.pone.0066901 PMC3689669

[B22] Solyman SM Black CC Duim B Perreten V van Duijkeren E Wagenaar JA Eberlein LC Sadeghi LN Videla R Bemis DA Kania SA Multilocus sequence typing for characterization of *Staphylococcus pseudintermedius* J Clin Microbiol 2013 51 306 310 23115265 10.1128/JCM.02421-12 PMC3536184

[B23] Paul NC Bärgman SC Moodley A Nielsen SS Guardabassi L *Staphylococcus pseudintermedius* colonization patterns and strain diversity in healthy dogs: a cross-sectional and longitudinal study Vet Microbiol 2012 160 420 427 22770516 10.1016/j.vetmic.2012.06.012

[B24] Damborg P Moodley A Guardabassi L American Society for Microbiology High genetic diversity among methicillin-resitant *Staphylococcus pseudintermedius* isolated from canine infection in Denmark Proceeding of the 3rd ASM-ESCMID Conference in Methicillin-resistant Staphylococci in Animals: 4–7 November, Copenhagen, Denmark 2013 80

[B25] Ben Zakour NL Bannoehr J van den Broek AH Thoday KL Fitzgerald JR Complete genome sequence of the canine pathogen *Staphylococcus pseudintermedius* J Bacteriol 2011 193 2363 2364 21398539 10.1128/JB.00137-11 PMC3133065

[B26] Guardabassi L Loeber ME Jacobson A Transmission of multiple antimicrobial-resistant *Staphylococcus intermedius* between dogs affected by deep pyoderma and their owners Vet Microbiol 2004 98 23 27 14738778 10.1016/j.vetmic.2003.09.021

[B27] Lu YF McEwan NA Staphylococcal and micrococcal adherence to canine and feline corneocytes: quantification using a simple adherence assay Vet Dermatol 2007 18 29 35 17222237 10.1111/j.1365-3164.2007.00567.x

[B28] Moodley A Espinosa-Gongora C Nielsen SS McCarthy AJ Lindsay JA Guardabassi L Comparative host specificity of human- and pig- associated *Staphylococcus aureus* clonal lineages PLoS One 2012 7 e49344 23166643 10.1371/journal.pone.0049344 PMC3498157

[B29] Quitoco IM Ramundo MS Silva-Carvalho MC Souza RR Beltrame CO de Oliveira TF Araújo R Del Peloso PF Coelho LR Figueiredo AM First report in South America of companion animal colonization by the USA1100 clone of community-acquired meticillin-resistant *Staphylococcus aureus* (ST30) and by the European clone of methicillin-resistant *Staphylococcus pseudintermedius* (ST71) BMC Res Notes 2013 6 336 23981343 10.1186/1756-0500-6-336 PMC3765899

[B30] Lowy FD *Staphylococcus aureus* infections N Engl J Med 1998 339 520 532 9709046 10.1056/NEJM199808203390806

[B31] Pöhlmann-Dietze P Ulrich M Kiser KB Döring G Lee JC Fournier JM Botzenhart K Wolz C Adherence of *Staphylococcus aureus* to endothelial cells: influence of capsular polysaccharide, global regulator agr, and bacterial growth phase Infect Immun 2000 68 4865 4871 10948098 10.1128/iai.68.9.4865-4871.2000 PMC101683

[B32] Ythier M Resch G Waridel P Panchaud A Gfeller A Majcherczyk P Quadroni M Moreillon P Proteomic and transcriptomic profiling of *Staphylococcus aureus* surface LPXTG-proteins: correlation with agr genotypes and adherence phenotypes Mol Cell Proteomics 2012 11 1123 1139 22843989 10.1074/mcp.M111.014191 PMC3494191

[B33] Georghegan JA Monk IR O’Gara JP Foster TJ Subdomains N2N3 of Fibronectin Binding Protein A mediate *Staphylooccus aureus* biofilm formation and adherence to fibrinogen using distinct mechanisms J Bacteriol 2013 195 2675 2683 23564165 10.1128/JB.02128-12 PMC3676058

[B34] Woolley KL Kelly RF Fazakerley J Williams NJ Nuttall TJ McEwan NA Reduced in vitro adherence of Staphylococcus species to feline corneocytes compared to canine and human corneocytes Vet Dermatol 2008 19 1 6 18177284 10.1111/j.1365-3164.2007.00649.x

[B35] Kadlec K Schwarz S Perreten V Andersson UG Finn M Greko C Moodley A Kania SA Frank LA Bemis DA Franco A Iurescia M Battisti A Duim B Wagenaar JA van Duijkeren E Weese JS Fitzgerald JR Rossano A Guardabassi L Molecular analysis of methicillin-resistant *Staphylococcus pseudintermedius* of feline origin from different European countries and North America J Antimicrob Chemother 2010 65 1826 1828 20534627 10.1093/jac/dkq203

[B36] Pilla R Bonura C Malvisi M Snel GG Piccinini R Methicillin-resistant *Staphylococcus pseudintermedius* as causative agent of dairy cow mastitis Vet Rec 2013 173 19 23723102 10.1136/vr.101485

[B37] McCarthy AJ Lindsay JA Loeffler A Are all meticillin-resistant *Staphylococcus aureus* (MRSA) equal in all hosts? Epidemiological and genetic comparison between animal and human MRSA Vet Dermatol 2012 23 267 275 22823579 10.1111/j.1365-3164.2012.01072.x

[B38] Weidenmaier C Kokai-Kun JF Kristian SA Chanturiya T Kalbacher H Gross M Nicholson G Neumeister B Mond JJ Peschel A Role of teichoic acids in *Staphylococcus aureus* nasal colonization, a major risk factor in nosocomial infections Nat Med 2004 10 243 245 14758355 10.1038/nm991

[B39] Paul NC Latronico F Moodley A Nielsen SS Damborg P Guardabassi L In vitro adherence of *Staphylococcus pseudintermedius* to canine corneocytes is influenced by colonization status of corneocytes donors Vet Res 2013 8 44 52 10.1186/1297-9716-44-52 PMC3716657 23834238

